# Interprofessional oral health promotion in rural primary healthcare systems: a scoping review

**DOI:** 10.3389/froh.2026.1958420

**Published:** 2026-09-23

**Authors:** Hlulani Alloy Nghayo, Khabiso Jemima Ramphoma, Ronel Deidre Maart

**Affiliations:** 1 Department of Rehabilitation and Dental Sciences, Faculty of Science, Tshwane University of Technology, Pretoria, South Africa; 2 Department of Community Dentistry, Faculty of Dentistry, University of the Western Cape, Cape Town, South Africa; 3 Department of Prosthodontics, Faculty of Dentistry, University of the Western Cape, Cape Town, South Africa

**Keywords:** health system integration, interprofessional collaboration, oral health promotion, primary healthcare, rural communities

## Abstract

**Objectives:**

This scoping review aimed to examine the literature on the design, integration, adaptation, and sustainability of interprofessional oral health promotion interventions (IOHPIs) within rural primary healthcare (PHC) systems.

**Methods:**

A scoping review was conducted following the Arksey and O'Malley framework. Five databases (EBSCOhost, MEDLINE, PubMed, ScienceDirect, and Wiley Online Library) and manual search were used to identify peer-reviewed articles published in English between January 2016 and February 2026. Studies were eligible if they described IOHPIs that had been implemented within rural PHC systems. Two reviewers independently screened records to ensure rigour and charted them across seven *a priori* domains: interprofessional model, workforce strategy, implementation, PHC integration, contextual adaptation, sustainability, and outcomes.

**Results:**

Of 2,899 records, 12 studies met the inclusion criteria. Eleven were conducted in high-income countries, while one originated from a low- and middle-income country. Four models of integration were identified: (1) clinic-embedded medical-dental integration, (2) public-service-hub integration, (3) Indigenous community-governed integration, and (4) telehealth-enabled outreach. In these models, preventive responsibilities were shared among multidisciplinary PHC teams, including nurses, community and Aboriginal health workers, hygienists, and therapists, under structured supervision. Outcomes were grouped into service access, preventive delivery, and implementation. Sustainability relied more on structural integration within workflows, financing, and governance than on clinical intent.

**Conclusion:**

The review indicates that IOHPIs are more likely to remain sustainable when preventive activities are firmly integrated into routine PHC systems and supported by shared workforce roles, local contextual adaptation, and enabling organisational processes. However, the evidence base remains small, geographically concentrated, and limited in terms of long-term, economic, and equity-oriented evaluation, particularly in low- and middle-income countries. Further implementation research is needed to determine whether these models can be sustained and scaled in diverse rural and low-resource settings.

## 1 Introduction

The World Health Organisation (WHO) has pioneered a global oral health initiative aligned with the Sustainable Development Goals (SDGs), with the aim of achieving universal health coverage (UHC) by prioritising access to essential, high-quality health services tailored to the specific needs of individuals and communities (1, 2). Despite these efforts, oral diseases remain a major global public health challenge, affecting approximately 3.7 billion people and contributing significantly to disability, pain, and a reduced quality of life. Notably, the burden of these diseases is unevenly distributed (3, 4). People living in socioeconomically disadvantaged, geographically isolated, and structurally underserved settings experience greater unmet needs, poorer access to preventive care, and more advanced disease at presentation (5, 6). These inequities are often compounded by workforce shortages, limited infrastructure, long travel distances, fragmented referral pathways, and restricted access to routine preventive care (5–7).

To address these inequalities, the WHO has advocated for the integration of oral health into primary healthcare (PHC) services, as PHC is central to providing safe, effective, affordable, and accessible essential oral healthcare (3, 8). PHC is widely recognised as a key platform for improving equity, prevention, and continuity of care across the life course. Within this framework, integrating oral health offers an important opportunity to empower individuals and bring prevention and early detection closer to the communities that need them most (9, 10). However, in many settings, oral health remains structurally separate from broader health and community systems (11, 12). This separation is particularly difficult to overcome in rural settings, where fragmented services, limited access to specialists, and organisational constraints make integrated care more challenging to establish and sustain (11, 13).

Furthermore, interprofessional collaboration (IPC) has therefore been endorsed as a practical, systems-level response to these gaps. IPC is a cooperative approach among healthcare professionals from different fields aimed at delivering services for the benefit of healthcare users (14, 15). Rather than relying solely on the oral health workforce, interprofessional approaches seek to distribute oral health responsibilities across multiple providers within existing PHC systems, including nurses, community health workers (CHWs), dental hygienists (DHs), oral health therapists (OHTs), and other medical professionals (9, 16, 17). In practice, this approach can support screening, counselling, referral, preventive treatment, and follow-up during routine care encounters. It also creates opportunities to integrate oral health within everyday PHC delivery rather than treating it as a separate service added on after the fact (18, 19). However, despite these established links and the growing body of research, oral healthcare remains fragmented. Oral health professionals tend to work in silos, separately from other healthcare professionals (20, 21), while oral health remains poorly integrated into PHC systems (22–24), particularly in low-resource settings (25, 26).

Although evidence suggests that integrated oral health interventions, including telehealth-supported supervision in geographically isolated settings, can improve the delivery of preventive services and referral processes, the literature remains fragmented across disciplines, populations, and settings (9, 27, 28). Existing reviews have often examined medical–dental integration, task-sharing, or school-based programmes separately, and many have not considered rural PHC systems as a distinct implementation context (16, 29). Consequently, there remains limited understanding of how interprofessional oral health promotion interventions (IOHPIs) are designed, integrated, adapted, and sustained within rural health services.

Given the growing evidence supporting the integration of oral health into PHC systems and the increasing emphasis on IPC as a means of achieving UHC, particularly in rural settings, there is a need to systematically map the breadth and depth of the available evidence and identify knowledge gaps. Accordingly, this scoping review aimed to systematically map IOHPIs implemented within rural PHC systems. Specifically, it examined: (i) interprofessional models; (ii) workforce strategies; (iii) implementation approaches; (iv) PHC integration mechanisms; (v) contextual adaptation; (vi) sustainability indicators; and (vii) reported outcomes. By characterising these analytical domains, the review seeks to clarify how interventions have been designed, integrated, adapted, and sustained within rural PHC systems.

## 2 Methods

### 2.1 Conceptualisation of rurality

In this review, rurality is understood not merely as a geographical classification, but as a service context shaped by workforce maldistribution, infrastructural limitations, weak referral networks, and restricted access to preventive oral health services (30, 31). This broader conceptualisation is important because rural communities often face overlapping structural barriers that influence both implementation and outcomes. Accordingly, the review focuses on interventions designed for, or adapted to, rural, remote, Indigenous, and other underserved PHC environments.

### 2.2 Frameworks and reporting standards

This scoping review was conducted in accordance with the five-stage framework proposed by Arksey and O'Malley (32) and refined by the Joanna Briggs Institute (JBI) (33). Reporting followed the Preferred Reporting Items for Systematic Reviews and Meta-Analyses extension for Scoping Reviews (PRISMA-ScR) checklist (34).

### 2.3 Stage 1: identifying the research questions

The Population–Concept–Context (PCC) framework (35) informed development of the research questions. The Population comprised rural populations; the Concept included implemented IOHPIs; and the Context was rural PHC systems.

The primary research question was:

- *What interprofessional interventions have been implemented to promote oral health within rural PHC systems?*

The following sub-questions were specified *a priori*:

- *What interprofessional models have been used?*
- *What workforce strategies have been employed?*
- *What implementation strategies have been reported?*
- *What PHC integration mechanisms have been described?*
- *How have interventions been adapted to the local context?*
- *What sustainability indicators have been reported?*
- *What outcomes have been documented?*

### 2.4 Stage 2: identifying relevant studies

A search strategy was developed in consultation with a university-based health science librarian. Five databases, namely EBSCOhost, MEDLINE, PubMed, ScienceDirect, and Wiley Online Library, were searched for peer-reviewed literature published in English between January 2016 and February 2026 to capture paradigm shifts and newly emerged interventions. Manual searches were also conducted. The search concepts were derived from the review aim and included oral health promotion, IPC, PHC, rural contexts, and implementation or integration. Controlled vocabulary, such as Medical Subject Headings (MeSH), and free-text terms were combined using the Boolean operators AND and OR. The full search strategy is provided in Supplementary File S1.

### 2.5 Stage 3: study selection

Eligibility criteria were defined using the PCC framework (Table 1). Studies were included if they reported on implemented IOHPIs integrated into rural PHC systems. Only peer-reviewed articles published in English between January 2016 and February 2026 were considered. All identified records were imported into EndNote™ 20 [Clarivate Analytics, United States of America (USA)] (36) for removal of duplicates. Two reviewers (HAN, KJR) independently screened titles and abstracts according to the predefined eligibility criteria. Studies deemed potentially suitable were retrieved in full text and assessed independently by all reviewers (HAN, KJR, RDM). Any disagreements were resolved through discussion and consensus.

**Table 1:** Inclusion and exclusion criteria.

Criteria	Inclusion	Exclusion
Population	Studies involving explicitly described rural, remote, underserved, disadvantaged, marginalised, vulnerable, Indigenous, Aboriginal, Tribal, low-resource, resource-limited, low-income, or hard-to-reach populations across the life course.	Urban or general populations without explicit rural or underserved contextual descriptors; studies in which contextual classification was inferred by reviewers rather than stated by the authors.
Concept	Implemented interprofessional oral health promotion approaches involving collaboration between two or more professional groups, characterised by shared planning, co-delivery, coordinated referral systems, or integrated service delivery within PHC contexts. Only studies reporting implemented interprofessional service delivery were eligible.	Perception-only studies; readiness, acceptability, or feasibility studies without an implemented intervention; editorials, commentaries, protocols, conference abstracts, narrative, or review studies; publications not subjected to peer review; policy reports, organisational guidelines, book chapters, dissertations, theses, and government documents; single-discipline interventions; and interprofessional education (IPE) studies reporting student learning outcomes without service implementation.
Context	Interventions integrated into rural PHC systems or PHC-linked public health service platforms (e.g., community health centres, mobile PHC services, or school-linked PHC programmes).	Programmes not integrated into PHC systems; infrastructure-level analyses without implemented service delivery; hospital-based specialist services without PHC integration; stand-alone outreach initiatives without PHC linkage.
Language	English-language publications.	Non-English publications.
Year of publication	January 2016 to February 2026.	Publications prior to January 2016 or after February 2026.

### 2.6 Stage 4: data extraction

A standardised data extraction form was co-developed by all reviewers. Data charting was iterative, with minor refinements made to the extraction form during the process. Data were extracted according to three categories:

- **Study characteristics** (author, year, country, aim or objective, study design, and population)
- **Interprofessional oral health integration domains specified *a priori*** (interprofessional model, workforce strategy, implementation strategy, PHC integration mechanisms, contextual adaptation, sustainability indicators)
- **Reported outcomes**

Two reviewers (HAN, KJR) independently extracted data from all included studies and cross-checked entries for accuracy. Any disagreements were resolved through discussion and consensus among all reviewers. In accordance with scoping review methodology, no formal assessment of the methodological quality of the included studies was conducted.

### 2.7 Stage 5: collating, summarising, and reporting the results

The study selection process is illustrated in a PRISMA-ScR flow diagram (see Figure 1). Extracted data were summarised descriptively and organised according to the predefined analytic domains. All reviewers examined and approved the categorisation of the findings prior to the final synthesis.

**Figure 1:**
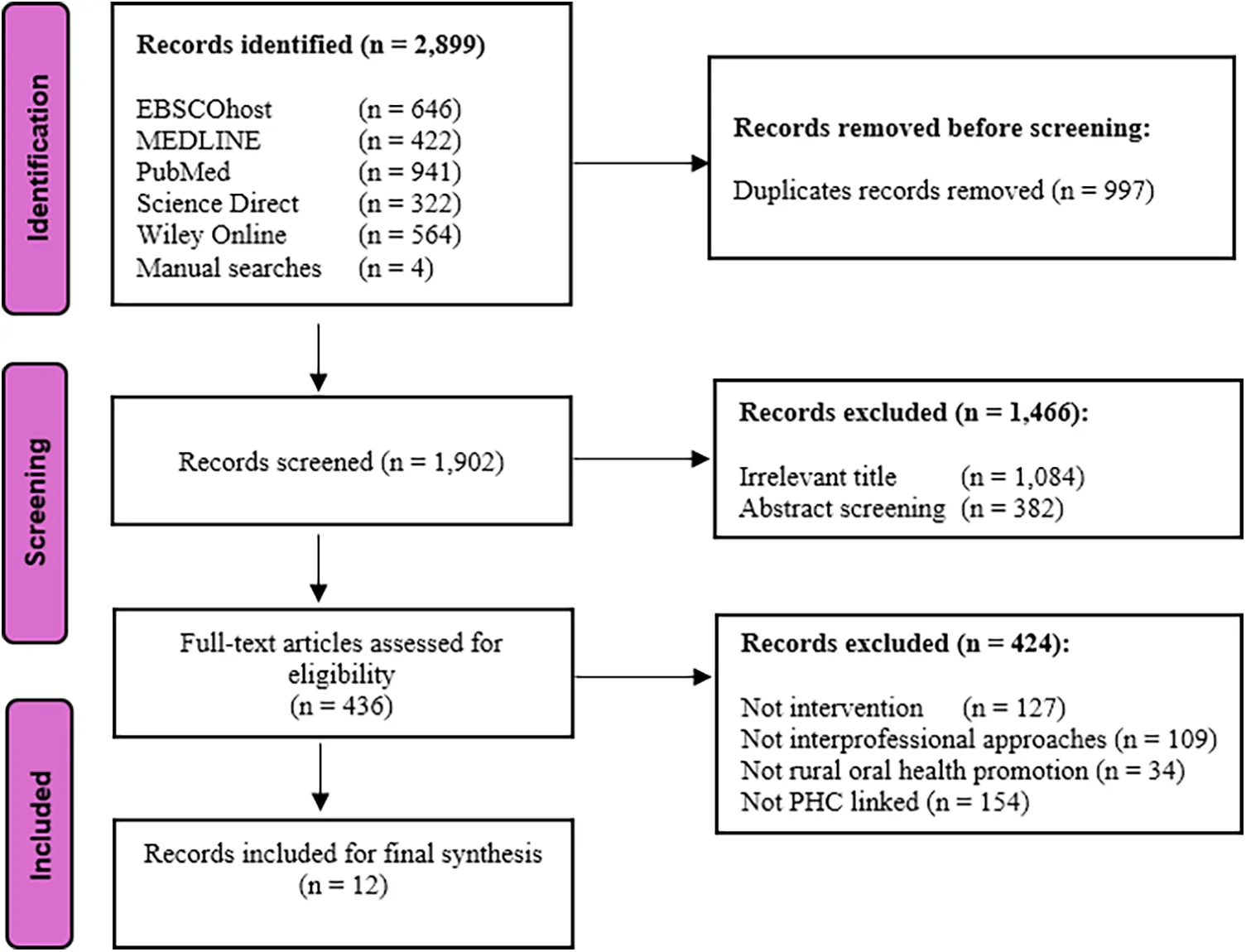
PRISMA flow diagram for the study selection process.

## 3 Results

The search yielded 2,895 records across five databases, with four additional records identified through other sources (Figure 1). After duplicates were removed, 1,902 records remained for title and abstract screening. Of these, 1,466 records were excluded, with 1,084 removed during title screening and 382 during abstract review. A total of 436 full-text articles were assessed for eligibility. Of these, 424 were excluded for the following reasons: not an intervention (*n* = 127), not interprofessional (*n* = 109), not related to rural oral health promotion (*n* = 34), and not linked to PHC (*n* = 154). Twelve studies (37–48) met the inclusion criteria and were included in the final synthesis. The characteristics of the included studies are summarised in Table 2.

**Table 2:** Characteristics of the included studies.

Author(s), Year	Country	Aim/Objective	Study Design	Population
Battani et al. 2023 (37)	USA	To describe a program that integrates dental hygienist (DHs) into obstetrics and gynecology (OB/GYN) clinics in federally qualified health centers (FQHCs) located in underserved areas of Michigan with the goal of improving the oral health of pregnant people.	Case study	Pregnant people
Braun et al. 2023 (38)	USA	To describe the implementation approach and evaluation of a medical-dental integration (MDI) project in Colorado that embedded dental hygienists (DHs) into 10 medical practice settings.	Case report	13,458 patient visits in 10 medical practices in Colorado (ages 0 to >65 years)
Braun et al. 2024 (39)	USA	To evaluate the effectiveness of *The Rocky Mountain Network for Oral Health Integration* (RoMoNOH) through 3 medical-dental integration (MDI) models medical visits by the medical team (model 1), by integrated DHs (model 2), or a hybrid of these models (model 3).	Pragmatic, multisite intervention study	Children aged 0 to 40 months
Clark et al. 2025 (40)	USA	To assess the impact of the Maternal and Infant Oral Health (MIMIOH) program on receipt of dental visits during pregnancy.	Pilot implementation	Women receiving prenatal care
Dooley et al. 2015 (41)	USA	To describe the process of improvement that took place after Contra Costa Regional Medical Center (CCRMC) partnered with the University of California San Francisco (UCSF) researchers to integrate oral health prevention into routine primary care practice.	Quality improvement	Children aged 1 through 5 years
Gold and Tomar 2018 (42)	USA	To describe the Women, Infants, and Children (WIC) Oral Health Program in a county in Florida and evaluate the needs for dental care.	Retrospective programme evaluation	576 children and 180 women
Kelly et al. 2024 (43)	Australia	To describes how First Nations *Kidney Warriors* (Aboriginal and Torres Strait Islander people living with kidney disease), dental hygienists, kidney health care professionals, an Aboriginal hostel accommodation manager and researchers co-designed an approach to improve oral health in South Australia.	Decolonising collaborative PAR	First Nations *Kidney* Warriors, DH students and interprofessional kidney care staff
Kong et al. 2021 (44)	Australia	To develop and pilot test the intervention, a culturally appropriate model of care with Aboriginal Health Workers to promote oral health during pregnancy; the model of care was named Grinnin' Up Mums & Bubs.	PAR	Seven Aboriginal Health Workers
Murphy and Larsson 2017 (45)	USA	To integrate and evaluate a pediatric oral health project in an American Indian, pediatric primary care setting.	Nonexperimental quality improvement	47 Caregiver/child dyads aged birth to 5 years
Tynan et al. 2018 (46)	Australia	To investigate the impact and experience of an integrated oral health program utilising tele-dentistry and Oral Health Therapists (OHT) in residential aged care facilities (RACFs) in a rural setting within Australia.	Mixed method comparison study	A total of 252 residents from nine regional and rural RACFs, including 27 residents who completed oral health-related quality-of-life surveys, and RACF staff.
Tynan et al. 2018 (47)	Australia	To describe the collaboration among the health services' Telehealth Department, Oral Health Team and residential aged care facilities (RACFs) staff that aimed to enable residents in rural and regional RACFs to have better access to oral health services.	Quality improvement	116 residents
Sampaio et al. 2021 (48)	Brazil	To evaluate the effectiveness of an integrated oral healthcare intervention for pregnant women and to analyse the association of sociodemographic, behavioural, oral health and general maternal and child health factors in prenatal care at usual risk in primary care in a city in the northeast of Brazil, in 2018–2019.	Controlled, randomised, single-blinded community trial	All pregnant women (146 in total, 58 from intervention group and 88 from control group)

DH, dental hygienist; FQHC, federally qualified health center; MDI, medical–dental integration; MIMIOH, Michigan Initiative for Maternal and Infant Oral Health; OB/GYN, obstetrics and gynaecology; PAR, participatory action research; RACF, residential aged care facility; WIC, Special Supplemental Nutrition Program for Women, Infants, and Children.

### 3.1 Study characteristics

Twelve studies met the inclusion criteria. Almost all were conducted in high-income countries (HICs) (37–47), with only one study undertaken in a low- and middle-income country (LMIC) (48). Most were conducted in the USA (*n* = 7) (37–42, 45), followed by Australia (*n* = 4) (43, 44, 46, 47), and Brazil (*n* = 1) (48).

Study designs were heterogeneous and included case studies and case reports (37, 38), pragmatic multi-site intervention studies (39), pilot implementation studies (40), quality improvement initiatives (41, 42, 47), retrospective programme evaluation (42), a mixed-method comparison study (46), participatory action research (PAR) (44), decolonising collaborative PAR (43), and one controlled, randomised, single-blinded community trial (48).

Populations included pregnant women and perinatal patients (37, 40, 42, 48), young children aged birth to five years of age (39, 41, 42, 45), and residents of regional and rural residential aged-care facilities (46, 47). Additional populations included First Nations peoples living with kidney disease (43), interprofessional health staff (43), and Aboriginal Health Workers participating in training and referral interventions (44). One large case report described 13,458 patient visits across 10 medical practices serving individuals aged from birth to over 65 years (38). Sample sizes ranged from small workforce or caregiver cohorts, such as seven Aboriginal Health Workers (44) and 47 caregiver–child dyads (45), to multisite or population-level evaluations involving more than 13,000 patient encounters (38). Table 3 summarises the key findings across the six domains of IOHPIs integrated within rural PHC systems, with descriptive details provided in Supplementary File S2.

**Table 3:** Interprofessional oral health integration domains.

Author(s), Year	Interprofessional Model	Workforce strategy	Implementation Strategy	PHC Integration	Contextual relevance	Sustainability indicators
Battani et al. 2023 (37)	DHs integrated into OB/GYN clinics within FQHCs	DHs embedded in prenatal teams; warm hand-offs; collaborative practice agreements	On-site dental operatories; structured workflows; staff training	Embedded in routine prenatal care; co-located clinics	Underserved populations; access barriers; 9 Michigan FQHC sites	Medicaid reimbursement; policy support; expansion to 9 sites
Braun et al. 2023 (38)	DHs integrated into primary care medical practices (MDI Learning Collaborative)	DH as care-team member; QI metric collection; referral to dentists	Startup–Implementation–Maintenance phases; 13-week training; practice coaches	Embedded in rural family medicine and OBGYN health centres	Low-income, Medicaid/uninsured, marginalised rural populations	Maintenance phase 2020–22; quarterly metrics; solvency review
Braun et al. 2024 (39)	Three MDI models: medical-provider-led, DH-embedded, and hybrid	Medical providers/DHs deliver CRA, FVA, referrals; practice facilitators	eLearning modules; Plan-Do-Study-Act cycles; Lean/Six Sigma; learning collaborative (4 states)	Preventive services in well-child visits; 54% co-located	Low-income, rural/frontier community health centres	4-year implementation; EHR integration; incentive payments
Clark et al. 2025 (40)	MIMIOH – dental hygienist embedded in OBGYN clinic	Registered DH coordinates with OBGYN staff; education each trimester	In-person/virtual trainings; regional coordinators; on-site dental space	Embedded FQHC obstetric clinic; prenatal-visit coordination	Low-income pregnant women; Medicaid; perinatal disparities	Funded by MDHHS/Delta Dental; visit rates declined post-funding
Dooley et al. 2015 (41)	Interdisciplinary safety-net academic partnership	Nurses apply fluoride varnish; RDHAP as project manager	Clinical policies; EHR prompts; Plan-Do-Study-Act cycles; stepwise dissemination	FV/education/referral in well-child visits (8 FQHC sites)	High ECC burden; low-income Medicaid; access disparities	95%–97% sustained FV rates; institutional commitment; >11,000 applications
Gold and Tomar, 2018 (42)	Non-traditional interdisciplinary programme at WIC offices	Dentist/DH provide education, screening, SDF; motivational interviewing	Weekly WIC-based clinic; one-stop model; recall aligned to WIC visits	Integrated into WIC programme; referral network	Low-income, underinsured, immigrant population; access barriers	Sustained 2013–16; Medicaid/grant funding; service-learning potential
Kelly et al. 2024 (43)	Kidney care–oral health working group (co-design)	DH student placement; Aboriginal Health Practitioner; renal nurses	Four-phase approach; cultural safety training; pilot to embedded placement	Embedded within kidney/dialysis services	First Nations peoples; colonisation, racism, rural relocation	Grant funding secured; cultural safety framework developed
Kong et al. 2021 (44)	Oral health promotion with culturally safe referral pathway	Aboriginal Health Workers trained to screen and refer	Brochures, training workshop, modified referral pathway; pre–post pilot	Embedded in existing antenatal services	Aboriginal/Torres Strait Islander pregnant women; access barriers	Recommended permanent integration; ongoing training/resource development
Murphy and Larsson, 2017 (45)	Interprofessional initiative in non-dental AI setting	Paediatric nurse practitioner screens/refers; dental team same day	Three-part intervention; OHRAT; same-day referral (∼4.7 min visits)	Combined well-child/well-dental same-day visits	AI community; high ECC; access/literacy barriers	Requires staff-time reallocation; FV integration under discussion
Tynan et al. 2018 (46)	Integrated oral health programme with OHT and tele-dentistry	Visiting OHT screens/educates; remote dentist via tele-dentistry	Rolled out to RACFs; comparative model; accreditation audits	Governed by public hospital/health service	Rural/regional RACFs; large distances to dentists	Improved accreditation compliance; described as efficient model
Tynan et al. 2018 (47)	Telehealth–OHT–RACF collaboration	OHT screens/educates; nurse liaison; remote dentist	Trial from Nov 2014; personalised care plans; cost analysis	Governed by public hospital/health service partnership	Rural/regional RACFs; travel and dementia-related barriers	Rolled out further; plan compliance improved 53%→96%
Sampaio et al. 2021 (48)	Integrated prenatal oral health care intervention	CHWs, dentists, nurses, nutrition/nursing students	Biweekly collective sessions; CHW home visits; risk stratification	Conducted within BHU primary/prenatal care	Northeastern Brazil; low income; socioeconomic determinants	Integrated into routine prenatal care; PHC infrastructure

AI, American Indian; BHU, basic health unit; CHW, community health worker; CRA, caries risk assessment; DH, dental hygienist; ECC, early childhood caries; EHR, electronic health record; FQHC, federally qualified health center; FVA, fluoride varnish application; MDI, medical–dental integration; MDHHS, Michigan Department of Health and Human Services; OBGYN, obstetrics and gynaecology; OHRAT, oral health risk assessment tool; OHT, oral health therapist; PHC, primary health care; RACF, residential aged care facility; RDHAP, registered dental hygienist in alternative practice; SDF, silver diamine fluoride; MIMIOH, Michigan Initiative for Maternal and Infant Oral Health; WIC, Special Supplemental Nutrition Program for Women, Infants, and Children.

### 3.2 Interprofessional oral health integration domains

#### 3.2.1 Interprofessional approaches

Across the included studies, four main models of integration were described: clinic-embedded medical–dental integration, public service–hub integration, Indigenous co-designed community integration, and outreach or telehealth-enabled service extension. The most frequently reported model involved integrating oral health services within routine primary medical care workflows, particularly within maternal, obstetric, and paediatric care platforms (37, 40, 41, 45). In these models, preventive oral health services were delivered during existing medical consultations. Variations included fully integrated medical–dental models and hybrid configurations supported by learning collaboratives and quality-improvement activities (38, 39).

A second model integrated oral health promotion within established public and community service hubs by aligning dental services with maternal and child health and social support platforms, such as WIC and Family Health Teams (42, 48). In Indigenous contexts, services were organised through co-designed, community-governed models that linked oral health with chronic disease care pathways (43, 44). In geographically constrained settings, outreach- and telehealth-supported approaches extended oral health services to residential aged-care facilities through collaboration among oral health services, telehealth units, and facility teams (46, 47).

#### 3.2.2 Workforce strategy

Workforce delivery involved redistributing preventive oral health functions across multidisciplinary PHC teams. The most commonly reported approach involved integrating DHs within medical settings under collaborative or indirect supervision arrangements (37, 38, 40). These arrangements enabled screening, preventive interventions, counselling, referral coordination, and documentation to be incorporated into routine care encounters. Hybrid models extended preventive oral health tasks to medical providers while retaining integrating dental personnel (39). Preventive activities were also delivered by non-dental PHC providers, including nurses applying fluoride varnish under standing orders (41), paediatric nurse practitioners conducting oral health screening and risk assessment (45), and medical providers undertaking caries risk assessments and applying fluoride varnish during consultations (39). Within community service platforms, dentists and DHs provided screening and preventive services while also training service staff (42).

In Indigenous and community-based settings, workforce strategies included training Aboriginal Health Workers to conduct screening and referrals using validated tools and structured referral pathways (44). In one study, an Aboriginal Kidney Warrior, a Kanggawodli manager and cultural safety educator, an AKction nurse researcher and lecturer, and an experienced DH collaboratively designed a responsive, decolonising PAR approach (43). Within Family Health Teams, CHWs conducted home visits for pregnant women at high risk, supported by dental and nursing follow-up (48). In rural settings with limited access to oral health services, telehealth-supported arrangements enabled visiting OHTs to conduct assessments and provide initial management under the remote supervision of dentists (46, 47).

#### 3.2.3 Implementation strategy

Implementation approaches included integrating oral health activities within routine PHC workflows, using structured roll-out processes, and establishing monitoring systems. In several studies, oral health services were incorporated into prenatal and well-child visits, supported by designated clinic space, referral pathways, and alignment with group care models (37, 40, 45). In maternal PHC settings, interventions included repeated group sessions, risk stratification, home visits, and appointment reminders integrated into antenatal care (48). Within community service hubs, preventive schedules and recall cycles were aligned with existing appointments (42). Several studies reported structured implementation processes, including clearly defined start-up, implementation, and maintenance phases; in-person and virtual training modules; and ongoing technical assistance (38–40, 44). Leadership engagement and clinician participation were also reported in two studies (37, 38).

Quality improvement activities were incorporated into several settings and included Plan–Do–Study–Act cycles, Lean Six Sigma approaches, electronic health record prompts, standing orders, billing protocols, and performance metric reporting systems (38, 39, 41). Financial incentives and grant funding were also reported as supporting implementation in some contexts (39, 43). In rural aged-care settings, implementation strategies included teledentistry-supported consultations, phased facility roll-out, and audits aligned with accreditation requirements (46, 47). In Indigenous contexts, studies reported phased co-design processes and the culturally adapted redesign of referral pathways (43, 44).

#### 3.2.4 PHC integration

PHC integration involved incorporating oral health services into existing clinical workflows, service environments, and referral systems. Preventive oral health services were delivered during established prenatal and well-child visits across FQHCs, community health centres, Basic Health Units, and Indian Health Service clinics (37, 39, 41, 45, 48). Integration also included incorporating oral health into discharge procedures and follow-up systems (40). The co-location of medical and dental services was reported in some studies, enabling direct referral pathways and same-day consultations (37, 45). In rural family medicine and community health settings, dental personnel participated in shared referral and care-coordination processes (38). Formalised referral networks were described within public oral health systems, including linkages with university clinics, Aboriginal Community Controlled Health Services, public dental services, and private providers (42, 44). Adjustments to booking systems and culturally adapted referral pathways were also reported in antenatal and Aboriginal health services (44). In chronic disease and aged-care settings, oral health services were coordinated with kidney health programmes, dialysis services, hospital networks, telehealth departments, and residential aged-care facilities (43, 46, 47).

#### 3.2.5 Contextual relevance

Contextual considerations included socioeconomic disadvantage, geographical access constraints, cultural safety, and local disease burden. Many interventions were implemented in community health centres and FQHCs serving low-income, Medicaid-insured, and uninsured populations (37–40). Studies described efforts to address barriers such as limited dental coverage, transport constraints, difficulties accessing services among immigrant populations, and long waiting times (41, 42). Several interventions responded to the high prevalence of early childhood caries in communities characterised by limited access to paediatric dental care, low oral health literacy, poverty-related constraints, and non-fluoridated water supplies (41, 45). In Indigenous contexts, studies reported culturally adapted approaches, including community consultation, culturally tailored educational resources, and attention to experiences of racism and marginalisation (43, 44). Interventions for Aboriginal and Torres Strait Islander pregnant women addressed cost, eligibility barriers, and limited access to public dental services (44). In rural aged-care settings, geographical dispersion, distance from providers, dementia-related care complexity, and staffing constraints influenced service delivery models (46, 47). Socioeconomic profiling of service areas was reported in one study (48).

#### 3.2.6 Sustainability indicators

Indicators of sustainability included continuation beyond the initial implementation period, integration into routine documentation systems, established financing mechanisms, and evidence of service expansion. Several studies reported the integration of oral health activities into electronic health record workflows, standing policies, staff onboarding processes, and routine documentation practices (39, 41, 48). Maintenance-phase performance monitoring and sustained service volumes were also described in some settings (38, 41). Medicaid reimbursement, incentive payments, grant funding, and institutional support were associated with continued service delivery in several studies (37, 39, 42). Expansion across sites, roll-out to additional facilities, and compliance with accreditation standards were reported as indicators of ongoing implementation (37, 46, 47). In other settings, interventions progressed from pilot implementation to routine service delivery (43). Some studies recommended incorporating oral health responsibilities into routine staff time allocations and service processes (44, 45). Conversely, declines in service utilisation following programme cessation were reported in some contexts (38, 40).

### 3.3 Key findings/outcomes

Table 4 summarises the reported outcomes. The outcomes were grouped into four categories: service access, preventive service delivery, clinical indicators, and system or workforce outcomes. Expanded access to oral health services was the most frequently reported outcome, particularly among pregnant women, young children, Indigenous communities, and residents of aged care facilities (37–40, 42, 43, 45, 46). Increased dental care utilisation during the implementation phases was reported (40), along with greater delivery of preventive services, such as fluoride varnish (39, 41). Sustained fluoride varnish application rates of 95% to 97% were reported in some safety-net PHC settings (41). Medical-dental integration was associated with reductions in untreated decay (38). Integrated prenatal oral healthcare was associated with improved oral health status and positive reported pregnancy outcomes (48). In residential aged care settings, improved oral health management and adherence to care plans were described (46, 47). Workforce-related outcomes included increased oral health knowledge and confidence among Aboriginal Health Workers (44), interprofessional education among aged care staff (47), and strengthened referral processes in First Nations communities (43). System-level outcomes included improved care coordination, reduced travel requirements, and more efficient use of existing PHC infrastructure (45–47).

**Table 4:** Summary of key findings/outcomes.

Author(s), Year	Key Findings/Outcomes
Battani et al. 2023 (37)	This programme demonstrated the potential for DHs to use collaborative practice agreements and remote supervision to increase access to oral health care for pregnant people. Integrating DHs into primary care settings increased access to oral health care, particularly for individuals who experienced difficulties accessing traditional oral health services. Allowing DHs greater autonomy to practise at the highest level within their scope of practice could improve access to oral health care for populations living in dentally underserved areas. The programme demonstrated that integrating DHs into obstetrics and gynaecology (OB/GYN) settings improved access to oral health care for pregnant people.
Braun et al. 2023 (38)	DHs integrated into medical teams provided full-scope dental hygiene care and expanded access to dental services. Integrating DHs into primary care medical practices has the potential to expand access to oral health care among marginalised populations and promote health equity. Medical-dental integration (MDI) care was variably associated with reductions in untreated dental decay.
Braun et al. 2024 (39)	MDI was associated with the greatest increase in the delivery of preventive oral health services (POHS). The intervention's alignment with fluoride varnish application (FVA) recommendations by medical providers for young children highlights its importance in advancing oral health care practices. The integration of DHs into medical teams has the potential to improve access to dental services.
Clark et al. 2025 (40)	The Michigan Initiative for Maternal and Infant Oral Health (MIMIOH) programme successfully increased rates of dental care among pregnant women during periods of active participation.
Dooley et al. 2015 (41)	This multidisciplinary team project demonstrated that oral health interventions in primary care, including routine topical FV applications, can be successfully integrated into early childhood well-child visits in safety-net primary care health centres. This team project developed care processes that fitted smoothly into the daily flow of well-child care, resulting in a sustained FV rate of 95% to 97% in the targeted age group. The intervention revealed the potential to bring about a significant reduction in ECC within a high-risk, diverse, safety-net population of young children.
Gold and Tomar 2018 (42)	Interdisciplinary collaboration between non-dental providers and oral health professionals is essential to improve the oral health of vulnerable populations. This programme demonstrates that collaboration with the WIC programme can improve access to oral health services for underserved populations. It delivers culturally appropriate education and health promotion for those at high risk of dental disease, while also providing preventive dental services and direct referrals to dental providers at no additional cost to patients. The programme also creates service-learning opportunities for students in dentistry, nursing, medicine, physician assistant studies and many other health programmes, thereby supplying additional support, logistical capacity and workforce for service delivery.
Kelly et al. 2024 (43)	This codesigned approach involving IPC and joint decision-making with community members has significantly informed improvements in oral health care information, services, and referral with and for First Nations Peoples with kidney disease. The project was effective in increasing access to oral health information, screening, and referral for First Nations Peoples. The project provided a practical example of how to decolonise health service and education programmes from the ground up.
Kong et al. 2021 (44)	The programme provided Aboriginal Health Workers with the knowledge and confidence to promote oral health and undertake dental referrals for pregnant women. Aboriginal Health Workers' reported a high level of confidence in discussing oral health care, screening pregnant women at risk of oral health problems, and referring clients to dental services after the training. Large improvements were reported in misconceptions about oral health during pregnancy, the link between oral health and general health, eligibility for access to public dental services, the safety of dental treatment during pregnancy, and early childhood oral health practices.
Murphy and Larsson 2017 (45)	The project's interprofessional education improved oral health for young, low-income children receiving oral health prevention services from a medical professional. This project improved access to oral health care for American Indian families showed how the design of healthcare facilities can promote health. The project's primary care providers were more cost-effective, feasible, and effective in ECC prevention and routine FVA.
Tynan et al. 2018 (46)	The integrated oral health programme has several potential benefits, including improving access to oral health education for aged care staff and preventive health care for residents, and minimising disruption to residents, especially those with high care needs, such as dementia. The integrated model helped to address inequality in access, as well as improving oral health education, promotion, disease prevention, and timely intervention. The integrated model, which incorporated OHTs and tele-dentistry, provides an efficient model that allows for improved relationships across services. The integrated oral health programme allowed referrals to a dental consultant to support locally based treatment, potentially reducing waiting lists and unnecessary travel.
Tynan et al. 2018 (47)	The programme assisted in improving oral health management and the potential to generate financial and social cost savings for residents and facilities. The programme allowed residents to be reviewed and treated in the comfort of a familiar environment, limiting the need for them to attend an oral health facility. It improved the interprofessional education of RACF staff, which has previously been described as important for improving the oral health of residents.
Sampaio et al. 2021 (48)	Integrated oral health care during prenatal care was associated with positive pregnancy and oral health outcomes. The involvement of multiple professionals in prenatal care contributed to improved self-care among pregnant women, with potential benefits for women's quality of life and future generations.

DH, dental hygienist; ECC, early childhood caries; FVA, fluoride varnish application; IPC, interprofessional collaboration; MDI, medical–dental integration; MIMIOH, Michigan Initiative for Maternal and Infant Oral Health; OHT, oral health therapist; OB/GYN, obstetrics and gynaecology; POHS, preventive oral health services; RACF, residential aged care facility; WIC, Special Supplemental Nutrition Program for Women, Infants, and Children.

## 4 Discussion

This scoping review mapped the existing evidence on the implementation of IOHPIs within rural PHC systems across the following *a priori* domains: interprofessional models, workforce strategies, implementation approaches, PHC integration mechanisms, contextual adaptation, sustainability indicators, and reported outcomes. It sought to clarify how these interventions are designed, integrated, adapted, and sustained in rural PHC settings. The review included and synthesised evidence from 12 IOHPIs (37–48), reflecting growing recognition of their importance within rural PHC systems. However, such interventions remain underrepresented particularly in rural LMIC communities. Overall, the findings indicate a clear pattern: rural oral health initiatives appear to be more sustainable when integrated into existing PHC structures rather than implemented as independent or isolated interventions. This suggests that the success of IOHPIs depends not only on the involvement of multiple professional disciplines, but also on the extent to which oral health is functionally integrated within routine care, organisational governance, and referral systems.

To strengthen the comprehensiveness and interpretive value of this scoping review, diverse range of study designs were included (Table 2), enabling the synthesis to capture both evaluative evidence and the practical realities of implementing IOHPIs within rural PHC systems. This broad scope aligns with the objectives of scoping review methodology, which is intended to map the breadth, characteristics, and contextual factors of the available evidence rather than focus solely on evaluating effectiveness (32). It enabled the review to capture not only formal intervention outcomes, but also the full pathway from intervention conceptualisation to sustained delivery in real-world settings. Although the evidence base was small and heterogeneous, the use of systematic search methods, multiple reviewers during screening, and independent data extraction enhanced the reliability and reproducibility of the findings. It is also important to note that no formal risk-of-bias assessment was conducted in this review.

Furthermore, an important finding was the limited availability of literature on the implementation of IOHPIs within rural PHC systems. This limitation is evident in the geographical concentration of the included studies, which predominantly originated from HICs, particularly the USA (37–42, 45) and Australia (43, 44, 46, 47). Only one study from an LMIC, conducted in Brazil, was identified (48). This geographical bias limits the generalisability and relevance of the findings to rural LMIC settings, where resource constraints, governance structures, workforce dynamics, and barriers to access may substantially influence IOHPI implementation (49). Although the implementation approaches identified in this review may have broader relevance, their applicability across diverse settings requires careful consideration and further implementation research (50). Consequently, while this review may contribute to a better conceptual understanding of the implementation of interprofessional interventions, it also points to the need for further evidence that is both geographically diverse and sensitive to rural contexts.

Although evidence relating to other rural populations and life stages remains limited in the current review, the findings highlight the growing global recognition of rural oral health, particularly through the integration of IOHPIs within rural PHC systems. This integration may help address the needs of groups with substantial preventive needs and regular contact with PHC, including pregnant women, perinatal patients, young children, and those using maternal and child health platforms for early intervention. Rural aged-care residents, who often experience mobility challenges and complex care needs, are also being considered in these initiatives, indicating a clear support for UHC in rural communities. These findings align with the SDGs and global initiatives that seek to integrate oral healthcare into PHC systems and adopt environmentally sustainable, preventive, and equitable practices to ensure that populations can access services without financial hardship (3, 51). The review further indicates recognition of the need to implement interventions designed to address structural inequities and promote culturally responsive care in rural communities. This approach is consistent with rural oral health reports emphasising the importance of developing contextually appropriate and culturally sensitive interventions that respond to the diverse needs of these communities (52, 53), thereby supporting a shift from conventional biomedical care towards more contextually relevant models of care.

The current review identified four distinct models for integrating IOHPIs within rural PHC systems: clinic-based medical–dental integration, public service hub integration, community integration co-designed with Indigenous groups, and service extension through outreach or telehealth. These models suggest that integrating oral health services within existing PHC platforms may offer implementation advantages than delivering them as separate interventions. Although their specific configurations vary by setting, all four models involve some form of structural integration, whether through routine clinical workflows, established community hubs, local governance structures, or technology-supported outreach. This suggests that rural oral health integration is not limited to a single model but encompasses a range of service arrangements shaped by context, workforce availability, and local system capacity. These findings align with a broader global health approach to oral health that emphasises integration into PHC, innovative workforce models responsive to population needs, people-centred care, tailored interventions across the life course, and the strategic use of digital technologies to strengthen oral health service delivery (3).

In response to the WHO's initiative to promote IPC as an innovative solution to the global health workforce crisis, rural PHC systems are reallocating roles by assigning oral health responsibilities to healthcare providers who are not traditional dental professionals (3). The findings of the current review are consistent with this global paradigm shift, indicating that workforce role redistribution is an emerging and defining feature of successful interprofessional oral health integration. Preventive responsibilities were shared across multidisciplinary PHC teams rather than concentrated solely among dentists, with nurses, CHWs, DHs, OHTs, and medical providers contributing to oral health service delivery in rural communities. These findings further illustrate that this transition has enabled the integration of screening, counselling, fluoride varnish application, referral coordination, and follow-up into routine PHC systems. In addition, task-sharing has emerged as an important approach because provider shortages and geographical isolation can undermine the sustainability of traditional specialist-led models in rural settings (54). Consistent with this, the findings of this review indicate that workforce redistribution, when supported by structured supervision and referral systems, may represent a practical and potentially sustainable approach to oral health promotion and increasing access to oral healthcare in rural communities.

The sustainability and effectiveness of oral health interventions are often undermined by weak structural integration, including limited institutionalisation, inadequate intersectoral collaboration, and discontinuity in political support (55). The current review reflects this gap, with a recurring finding that sustainability depends as much on structural integration as on clinical intent. Where oral health promotion was integrated into routine maternal, child, or community health encounters, it became part of everyday service delivery rather than a separate initiative requiring exceptional effort to sustain. Similarly, integrating oral health within clinical workflows, documentation systems, standing orders, and financing arrangements helped shift prevention from an occasional add-on to a normalised component of care. This aligns with previous research suggesting that the sustainability of oral health interventions may be strengthened when they are integrated within PHC systems rather than treated as an additional component (16). The current review extends this understanding by suggesting that, in rural settings, integration involves not only service design but also the organisation of systems to address challenges such as workforce shortages, geographical remoteness, and institutional instability.

The findings of this review further suggest that context played an active role in shaping the interventions by influencing their design, implementation, and perceived validity, rather than serving merely as a background setting. Although nearly all the studies were conducted in HICs, the interventions were implemented in low-income, underserved, and geographically isolated communities. These settings faced transport barriers, limited dental coverage, long waiting times, and high levels of unmet need, all of which restricted access to interprofessional healthcare. This suggests that rurality remains a significant global determinant of poor oral health outcomes and is frequently overlooked in the development of oral health policies (31). The review also indicated that interventions were strengthened when they were co-designed with communities and aligned with broader priorities such as chronic disease management, cultural safety, and local ownership. These approaches are important because they indicate that rurality is not merely a geographical descriptor, but a structural condition that influences which oral health interventions can be implemented, sustained, and trusted (31). The findings therefore imply that robust interventions were not only applicable in clinical settings but were also responsive to the sociopolitical dynamics of the communities they sought to serve.

Although further studies are necessary to understand the impact of telehealth in rural communities, its integration has been recognised as an intervention that may facilitate community engagement, facilitate outreach, and provide a dependable approach in rural communities (56). These findings are consistent with those of this study, which identified the value of PHC integration mechanisms through technology-supported approaches. For example, in geographically constrained settings, telehealth-enabled supervision allowed specialist input to be extended without requiring constant in-person specialist presence. In special care facilities, this model supported oral health oversight in environments where staffing constraints, geographical distance, and complex care needs often make routine dental access difficult. Although digital support cannot replace local clinical capacity (57), the findings of this study suggest that it may help strengthen service continuity when integrated into existing care pathways rather than used as a detached technological fix. The importance of this mechanism may lie not in its novelty, but in its capacity to enhance the accessibility, coordination, and responsiveness of care within constrained rural systems.

The current scoping review identified a persistent gap in the literature on the implementation, evaluation, and impact of IOHPIs in rural communities. The gap also reflects the need for a clearer articulation of shared professional roles and a stronger conceptualisation of interprofessional oral health integration. Most studies have focused on service access, preventive delivery, and implementation outcomes (37, 40, 42, 43, 45, 46), while fewer have examined clinical effects, equity impact, or cost-effectiveness (38, 39, 41, 45, 47, 48). Consequently, improved service reach may not necessarily translate into reduced oral health inequities or sustainable improvements in population health. Although several studies have reported better uptake, stronger referral pathways, and increased fluoride varnish delivery, relatively few have followed interventions long enough to determine whether these gains were sustained or whether they altered the broader distribution of oral health outcomes. The review findings are promising and indicate feasibility and potential across various rural settings; however, they do not conclusively demonstrate large-scale effectiveness, given the inclusion of descriptive and uncontrolled studies, as well as the geographic origin of the included studies.

The findings indicate that interprofessional oral health integration should be conceptualised as an issue of systems and governance, rather than merely one of workforce reorganisation. The models described across the mapped evidence commonly integrated oral health promotion into routine PHC operations, redistributed responsibilities across teams, and addressed local structural conditions. In policy and practice, strengthening rural oral healthcare requires more than isolated pilot projects or the enthusiasm of individual advocates; it calls for stable financing, shared accountability, clear supervision, and service designs that are structurally integrated into PHC. PHC managers can integrate oral health initiatives into existing facility workplans and earmark a portion of existing budgets for these activities. For example, by defining responsibilities through job descriptions and referral protocols, accountability can be enhanced among various staff groups. To track progress, a few indicators, such as community education sessions, basic screenings, and completed referrals can be added to the existing reporting systems. Implementation can be supported through supervisory guidance, continuous professional development, and recognition of teams or facilities that achieve integration objectives. These efforts can utilise existing PHC structures and funding without the need for separate oral health programmes. For research, the current priority is to move beyond proof-of-concept studies towards longitudinal, equity-focused, and economic evaluations that can show whether integration produces sustainable benefits in underserved rural and low-resource settings.

This review has several strengths. It followed the Arksey and O'Malley framework, refined by the JBI, and was reported in accordance with PRISMA-ScR guidelines, strengthening methodological transparency and reproducibility. Dual independent title and abstract screening and data extraction, independent full-text assessment by three reviewers, and a structured domain-based synthesis provided a robust and coherent account of how rural IOHPIs have been implemented. The broad analytical framing also enabled the review to go beyond simple programme description and develop a more systems-level understanding of integration, sustainability, and contextual adaptation. Several limitations should also be acknowledged. As is standard in scoping reviews, no formal critical appraisal of study quality was undertaken. The included studies were heterogeneous in design, setting, and outcome reporting, which limited the ability to draw causal inferences about effectiveness. Restricting the search to English-language, peer-reviewed studies may have excluded relevant grey literature and non-English implementation experiences, and the concentration of evidence in HICs reduces generalisability to rural LMICs. In addition, limited longitudinal reporting makes it difficult to determine whether the reported improvements were sustained over time.

## 5 Conclusion

This scoping review suggest that IOHPIs are more sustainable when they are structurally integrated within routine PHC systems and supported by shared workforce roles, reliable funding, and adaptation to local contexts. Although global policy frameworks continue to emphasise universal oral health coverage and alignment with the SDGs, the findings suggest that meaningful integration remains limited in LMICs, especially in Africa. The limited evidence on long-term outcomes, economic viability, and equity impacts highlights the need for stronger implementation research, particularly in rural communities within LMICs. The findings also emphasise the importance of moving beyond policy intent towards service models that are operationally and financially integrated within PHC systems. Future research should prioritise longitudinal, economic, and equity-oriented evaluations to determine whether such models can be sustained and scaled across diverse rural contexts.

## Data Availability

The original contributions presented in the study are included in the article/Supplementary Material, further inquiries can be directed to the corresponding author/s.
