# Supplementary material for: Interprofessional oral health promotion in rural primary healthcare systems: a scoping review
Source: Front Oral Health. 2026 Sep 23;7:1958420. doi: 10.3389/froh.2026.1958420 (PMC13642116; doi:10.3389/froh.2026.1958420)
Supplement: Supplementary file 1 [file Table1.docx]

Supplementary file 1: Search strategy

| **Database** | **Search string** | **Records** |
| --- | --- | --- |
| Pubmed 05/02/2026 | ("mouth"[MeSH Terms] OR Oral[Text Word]) OR ("dental health services"[MeSH Terms] OR Dental[Text Word]) AND (("health"[MeSH Terms] OR Health[Text Word]) OR ("delivery of health care"[MeSH Terms] OR Healthcare[Text Word])) AND (Promotion[All Fields] OR ("prevention and control"[Subheading] OR Prevention[Text Word]) OR ("education"[Subheading] OR "educational status"[MeSH Terms] OR "education"[MeSH Terms] OR Education[Text Word]) OR ("literacy"[MeSH Terms] OR Literacy[Text Word]) OR ("communication"[MeSH Terms] OR Communication[Text Word])) AND (Interprofessional[All Fields] OR ("interdisciplinary studies"[MeSH Terms] OR Interdisciplinary[Text Word]) OR ("interdisciplinary studies"[MeSH Terms] OR Multidisciplinary[Text Word]) OR Collaborative[All Fields] OR Integrated[All Fields] OR ("task shifting"[MeSH Terms] OR task-shifting[Text Word]) OR Intersectoral[All Fields] OR (Cross[All Fields] AND sector[All Fields])) AND (Primary[All Fields] OR PHC[All Fields] OR care[All Fields] OR Frontline[All Fields]) AND Rural[All Fields] OR Remote[All Fields] OR Underserved[All Fields] OR ("vulnerable populations"[MeSH Terms] OR Disadvantaged[Text Word]) OR Marginalized[All Fields] OR Vulnerable[All Fields] OR Indigenous[All Fields] OR Aboriginal[All Fields] OR Tribal[All Fields] OR Low-resource[All Fields] OR Resource-limited[All Fields] OR ("poverty"[MeSH Terms] OR Low-income[Text Word]) OR (("hardness"[MeSH Terms] OR Hard[Text Word]) AND reach[All Fields]) | 941 |
| EBSCOhost  05/02/2026 | (Oral OR Dental) AND (Health OR Healthcare) AND (Promotion OR Prevention OR Education OR Literacy OR Communication) AND (Interprofessional OR Interdisciplinary OR Multidisciplinary OR Collaborative OR Integrated OR task-shifting OR Intersectoral OR Cross-sector) AND (Primary OR PHC OR care OR Frontline) AND (Rural OR Remote OR Underserved OR Disadvantaged OR Marginalized OR Vulnerable OR Indigenous OR Aboriginal OR Tribal OR Low-resource OR Resource-limited OR Low-income OR Hard-to-reach) | 631 |
| Web of Science  05/02/2026 | ALL=((Oral OR Dental) AND (Health OR Healthcare) AND (Promotion OR Prevention OR Education OR Literacy OR Communication) AND (Interprofessional OR Interdisciplinary OR Multidisciplinary OR Collaborative OR Integrated OR task-shifting OR Intersectoral OR Cross-sector) AND (Primary OR PHC OR care OR Frontline) AND (Rural OR Remote OR Underserved OR Disadvantaged OR Marginalized OR Vulnerable OR Indigenous OR Aboriginal OR Tribal OR Low-resource OR Resource-limited OR Low-income OR Hard-to-reach)) and Article (Document Types) and Article (Document Types) and English (Languages) and All Open Access (Open Access) and All Open Access (Open Access) | 564 |
| Science direct  05/02/2026 | (Oral OR Dental) AND (Health) AND (Promotion) AND (Interprofessional OR Collaborative ) AND (Rural OR Remote OR Underserved) | 322 |
| Medline 05/02/2026 | (Oral OR Dental) AND (Health OR Healthcare) AND (Promotion OR Prevention OR Education OR Literacy OR Communication) AND (Interprofessional OR Interdisciplinary OR Multidisciplinary OR Collaborative OR Integrated OR task-shifting OR Intersectoral OR Cross-sector) AND (Primary OR PHC OR care OR Frontline) AND (Rural OR Remote OR Underserved OR Disadvantaged OR Marginalized OR Vulnerable OR Indigenous OR Aboriginal OR Tribal OR Low-resource OR Resource-limited OR Low-income OR Hard-to-reach) | 422 |
