# Supplementary material for: Interprofessional oral health promotion in rural primary healthcare systems: a scoping review
Source: Front Oral Health. 2026 Sep 23;7:1958420. doi: 10.3389/froh.2026.1958420 (PMC13642116; doi:10.3389/froh.2026.1958420)
Supplement: Supplementary file 2 [file Table2.docx]

**Supplementary File 2**: Descriptive details of Interprofessional oral health integration domains

| **Author(s), Year** | **Interprofessional Model** | **Workforce strategy** | **Implementation Strategy** | **PHC Integration** | **Contextual relevance** | **Sustainability indicators** |
| --- | --- | --- | --- | --- | --- | --- |
| Battani et al., 2023 [37] | Integration of full-time DHs into OB/GYN clinics, working collaboratively with prenatal care clinicians, administrators, and dental teams within FQHCs. | DHs embedded within prenatal care teams to provide oral health screening, preventive interventions, education, counselling, referrals, and warm hand-offs to dental clinics, enabled by collaborative practice agreements and indirect supervision. | Installation of dental operatories within OB/GYN clinics; structured yet flexible workflows; oral health presented as a standard part of prenatal care; training and buy-in of prenatal care professionals; and integration into routine and group prenatal visits, such as Centring Pregnancy. | Oral health care embedded within routine prenatal services at FQHCs, with the co-location of OB/GYN and dental clinics enabling referral pathways and continuity of care. | Focus on underserved populations facing barriers to traditional dental care; adaptation to clinic workflows, patient preferences, and socioeconomic constraints; implementation in geographically diverse FQHC sites across Michigan. | Medicaid reimbursement; collaborative practice policy (PA 161 Programme); leadership buy-in; co-location of services; expansion from six to nine FQHC sites; continued implementation at three sites post-COVID-19. |
| Braun et al., 2023 [38] | “Dental hygienists were integrated into primary care medical practices to provide full-scope dental hygiene care”; MDI Learning Collaborative; team-based care strategies; dental hygienist member of care team; engaged leaders/providers; data-driven quality improvement | DHs embedded in medical practices; trained to collect quality-improvement metrics; referred patients with restorative needs to partnering dentists; collaborative practice; full-scope dental hygiene care. | Startup, Implementation, Maintenance phases; 13-week training; practice transformation coaches; quality improvement approach; metric reporting via REDCap; learning collaborative meetings every six months; leadership and provider buy-in. | DHs embedded into primary care medical practices; rural family medicine community health centres; OBGYN community health centre; team-based care strategies; integrated workflow; shared care processes. | Low-income patients; Medicaid and uninsured populations; community health centres; rural family medicine sites; focus on marginalised populations. | Maintenance phase (spring 2020–2022); quarterly metric review; reduction in coaching; financial solvency themes (insured patients, encounter rate); evaluation of model; dissemination plans. |
| Braun et al., 2024 [39] | Three MDI models: (1) POHS delivered by medical providers; (2) POHS delivered by DHs embedded in the medical team; and (3) hybrid model; team-based care strategies; learning collaborative; practice facilitators. | Medical providers delivering CRA, FVA, referrals, goal setting; DHs embedded in medical team; hybrid models; practice facilitators providing coaching; IPC. | MDI change package; provider eLearning modules; monthly coaching; Plan-Do-Study-Act cycles; Lean and Six Sigma approaches; REDCap metric submission; financial incentives; learning collaborative across 4 states. | Preventive oral health services delivered during primary well-child care visits in community health centres; integration into routine medical workflow; co-located clinics (54%); embedded DH model; hybrid care models. | Community health centres serving low-income families; marginalised and underserved populations; rural and frontier sites; geographic disparities addressed. | 4-year implementation; system changes (electronic health records optimisation, onboarding training, policy modifications); incentive benchmark payments; sustainability criteria; continuation beyond baseline phase |
| Clark et al., 2025 [40] | “Michigan Initiative for Maternal and Infant Oral Health (MIMIOH)” placing a registered dental hygienist within an OBGYN medical clinic; collaboration between dental and obstetric providers; dental hygienist to meet with eligible patient during each trimester | Registered dental hygienist embedded in obstetric clinic; coordinate with OBGYN staff; provide oral health education and assessment during prenatal visits; schedule dental visits with on-site dental clinic; collaborative practice under PA-161 designation | In-person trainings for dental and OBGYN providers; monthly site visits by regional coordinators; monthly virtual meetings; designated dental operatory space within OBGYN clinic; technical assistance; programme timelines by cohort. | Embedded dental hygienist within FQHC obstetric clinic; coordination between medical and dental clinics; integration into prenatal visits; attribution of deliveries to FQHC sites | Low-income pregnant women; Medicaid coverage; FQHCs serving underserved communities; perinatal oral health disparities | Active participation periods; funding through MDHHS and Delta Dental Foundation; financial sustainability challenges noted; decline in visit rates after programme participation ended |
| Dooley et al., 2015 [41] | “Interdisciplinary safety net-academic partnership”; paediatrician, ambulatory nurse, quality nurse, dentist, dental hygienist (UCSF), registered dental hygienist in alternative practice (RDHAP); integration of oral health education, routine FV application, and referral into well-child visits. | Nurses applied fluoride varnish; providers ordered FV; standing nursing orders; RDHAP hired as project manager; collaboration with dental department; delegation of oral health tasks across care team. | Development of clinical policies; EPIC EHR prompts; integration into intake questionnaires; hands-on and didactic trainings; Plan-Do-Study-Act cycles; comparative feedback to clinics; billing protocol; standing orders; pilot then stepwise dissemination. | “Routine FV applications, along with oral health education, screening, and referral during well-child exams”; embedded across 8 FQHC primary care sites; integrated into routine workflow and discharge procedures. | High early childhood caries (ECC) burden; limited paediatric dental access; low-income Medicaid population; safety-net health system; racial/ethnic disparities; long wait times for dental care. | Sustained FV rates 95–97% (2012–2014); EHR integration; standing nursing policy; institutional commitment; annual >11,000 FV applications; integration across all 8 sites. |
| Gold & Tomar (2018) [42] | “Non-traditional interdisciplinary program” providing oral health education, dental screenings, preventive dental services, and referrals at WIC offices; collaboration between dental professionals (dentist/dental hygienist) and WIC staff; integration into federal WIC programme service hubs. | Dentist or dental hygienist provided education, screening, caries risk assessment, fluoride varnish and silver diamine fluoride (SDF) treatments; oral health education for WIC staff during monthly meetings; referrals to university paediatric clinic and rural medical/dental clinic. | Operated one day per week at WIC offices; “one-stop healthcare model”; oral health education delivered using motivational interviewing; fluoride varnish every 3 months for moderate/high risk children; SDF biannually when needed; recall appointments aligned with WIC visits. | Integrated into WIC public health programme; referrals to university paediatric dentistry clinic and local rural medical and dental clinic; list of Medicaid/low-fee providers provided; collaboration between oral health professionals and health service providers. | Low-income, underinsured, Medicaid-dependent population; high unmet dental needs; limited Medicaid coverage for pregnant women over 21 years; immigrant population barriers; transportation and access challenges. | Sustained from 2013–2016; supported by Medicaid reimbursements, grant funding, and institutional university support; potential for service-learning integration for dental, nursing, medical, PA students. |
| Kelly et al., 2024 [43] | Kidney care–oral health working group comprising First Nations Kidney Warriors, DHs, renal nurses, Aboriginal Health Practitioner, hostel manager, educators and researchers; co-design and joint decision making. | Dental hygienist student clinical placement at Kanggawodli; oral health promotion, screening and referral; interprofessional skills day; cultural safety and clinical yarning training; Aboriginal Kidney Warriors positioned as educators. | Four phases: (1) identification of oral health priorities; (2) cultural safety and interprofessional training; (3) student clinical placement pilot and expansion; (4) review and framework development; Australian Dental Health Foundation funding to support screening consumables. | Activities embedded within kidney health services and dialysis support at Kanggawodli; improved referral pathways; integration with renal services and Aboriginal health services. | First Nations Peoples experiencing colonisation, racism, marginalisation; rural and remote relocation for dialysis; cultural safety, Dadirri deep listening, Ganma knowledge sharing. | Clinical placement moved from pilot to embedded placement; plans for outreach dental clinic at Kanggawodli; secured grant funding; development of new kidney health–oral health cultural safety framework. |
| Kong et al., 2021 [44] | Model included “oral health promotion resources, training workshop, and a culturally safe referral pathway to dental services”; collaboration between Aboriginal Health Workers, researchers, public oral health services, and Aboriginal community stakeholders; training delivered by Aboriginal researchers and a researcher with a dental background. | Aboriginal Health Workers trained to “yarn, screen and refer clients (where appropriate) to the dental service”; use of a validated screening tool; provision of oral health promotion resources; Aboriginal dental assistant involved in referral pathway follow-up. | Development of brochure, fridge magnets, whiteboard educational tool, training workbook; face-to-face training workshop (1–2 hours); incorporation of screening tool; implementation of modified culturally safe referral pathway (direct call/email/QR code system); pilot using pre–post evaluation. | Implemented within existing antenatal service; referral pathways included public dental clinics, Aboriginal Community Controlled Health Services (ACCHSs), and private services; collaboration with South Western Sydney Local Health District Oral Health Service; adjustment of booking process to improve cultural safety. | Designed specifically for Aboriginal and Torres Strait Islander pregnant women; culturally tailored resources developed with Aboriginal graphic designer and community consultation; addressed barriers including cost, racism, eligibility requirements, and access to public dental services. | Recommendation for “permanent integration of the model into the existing antenatal service”; discussion of restructuring service to integrate training, resource printing, and referral pathways; suggestion to include training in Aboriginal Health Worker curricula; emphasis on PAR for sustainable translation. |
| Murphy & Larsson (2017) [45] | “Interprofessional oral health initiative in a nondental, AI setting”; collaboration between paediatric primary care provider and dental clinic “across the hall”; supervising dentist confirmed clinical findings and endorsed referral decisions; integration of well-child and well-dental visits into a combined same-day event. | Paediatric nurse practitioner completed oral health screening, caries risk assessment (OHRAT), education, and referral; dental team included “three general full-time dentists, one endodontist, one contract general dentist, one paediatric part-time dentist, one part-time oral surgeon, one dental hygienist… and eight dental assistants”; same-day referral to dental clinic. | Three-part intervention: (1) caregiver education; (2) caries risk assessment using OHRAT; (3) same-day dental home referral; “knee-to-knee oral health screening”; referral tracking slips; integration into routine well-child visits; visit duration mean 4.73 minutes. | Implemented within Indian Health Service paediatric primary care clinic; dental clinic located “across the hall”; referral process integrated into well-child visits; same-day well-dental visit offered; collaboration facilitated between medical and dental services. | AI reservation community; high prevalence of ECC; barriers include poverty, limited access, poor oral health literacy; nondental AI primary care setting; community water supply not fluoridated. | Authors state that sustaining the project would involve “reallocation of staff time to integrate oral health screening, education, and referral into well-child visits”; discussion of routine fluoride varnish integration; emphasis on incorporation into routine well-child care. |
| Tynan et al., 2018 [46] | “An integrated oral health program incorporating an OHT and tele-dentistry”; “multidisciplinary approach”; visiting OHT for screening, education, referral to a Dentist for remote real-time oral examination; RACF staff and significant others able to participate; collaboration between RACF, oral health service, and tele-health team. | Visiting OHT conducting screening, education, development of care plans; live-stream tele-dentistry consultations with remotely located Dentist; RACF staff responsible for oral health portfolio; OHT trained in intraoral camera manipulation; Dentist provides remote advice and review. | Programme “rolled out to RACFs within a hospital and health service”; comparative model (facilities with and without programme); OHT visits facilities; tele-dentistry used for remote screening, consultation, and treatment planning; audits aligned with Australian Aged Care Quality Accreditation Standards; integration with existing public oral health service. | Facilities governed by public hospital and health service; integration between RACFs, oral health service, and tele-health team; referral pathway to dentist via tele-dentistry; residents seen within familiar RACF environment; “collaborative approach between the oral health service, RACF and tele-health team”. | Rural and regional RACFs; large geographical health district (85,854 km²); access to nearest public dentist ranged from 5 km to 210 km; barriers included transport, dementia, limited staff skills; designed to address rural access inequities. | Improved compliance with accreditation standards; improved access to education and preventative care; reduction in need for travel; integrated within public health service; ongoing OHT support; described as “potentially efficient model” improving relationships across services. |
| Tynan et al., 2018 [47] | “Collaboration among the health services’ Telehealth Department, Oral Health Team and RACF staff”; model incorporated visit by an OHT for screening, simple intervention and referral for teledentistry session if required. | Visiting OHT conducted “screening, education, simple intervention and referral”; nurse allocated to oral health portfolio at each RACF; dentist provided remote advice via teledentistry; OHT operated intraoral camera and transmitted live feed to dentist. | Trial commenced November 2014; OHT employed to conduct screenings and oral health promotion activities; personalised oral health care plans placed in resident bathrooms; tele-dentistry used for review when required; audits conducted pre- and post-introduction; cost analysis performed. | Facilities governed by public hospital and health service; partnership between Telehealth Service, Oral Health Team and RACFs; referral to dentist via teledentistry; residents managed within RACFs to reduce need for transport to oral health facility. | Rural and regional RACFs; large geographical district; distance to nearest public dentist ranged from 4.6 km to 210 km; barriers included travel distance, dementia, ambulance transport; designed to improve access in regional and rural settings. | Programme being rolled out to other RACFs within health service; improved compliance with oral health care plans (53% to 96%); reduction in need for facility attendance; cost analysis performed; described as integrated model within health service. |
| Sampaio et al., 2021 [48] | “Integrated care intervention for pregnant women… within the scope of primary care”; dental treatment plan at BHU; “collective actions” using conversation wheel technique; involvement of dentists, nurses, nutrition students, CHWs, Family Health Teams. | Pregnant women classified as intermediate/high oral health risk received “a home visit from a community health worker (CHW)”; dental treatment registered in medical records; dentists conducted oral examinations; nurses involved in follow-up; research assistants (nutrition and nursing students) trained; Family Health Teams with dentists. | Biweekly “collective actions” (14 sessions across trimesters) addressing oral hygiene, nutrition, smoking, alcohol, breastfeeding, baby oral health; CHW home visits for high-risk cases; dental treatment plan at Basic Health Units (BHU); telephone reminders before sessions; risk stratification conducted at baseline and puerperal visit; integrated into prenatal routine at BHU. | Conducted within BHUs offering primary healthcare; family health teams including dentists; prenatal consultations integrated with dental care; medical records used; CHW involvement; routine prenatal follow-up within PHC structure. | Municipality in northeastern Brazil; MHDI 0.694; low family income (≤1 wage for 60.3%); integration into public health system; focus on socioeconomic and behavioural determinants; primary care setting serving vulnerable population. | Integrated into routine prenatal care at BHUs; risk stratification included in medical record; involvement of Family Health Teams; intervention delivered within existing PHC infrastructure; emphasis on network of dental services including primary care. |

**Abbreviations**: ACCHS, Aboriginal Community Controlled Health Service; AI, American Indian; BHU, Basic Health Unit; CHW, Community Health Worker; CRA, Caries Risk Assessment; DH, Dental Hygienist; ECC, Early Childhood Caries; EHR, Electronic Health Record; FQHC, Federally Qualified Health Center; FVA, Fluoride Varnish Application; IPC, Interprofessional Collaboration; MDI, Medical–Dental Integration; MDHHS, Michigan Department of Health and Human Services; OBGYN, Obstetrics and Gynaecology; OHRAT, Oral Health Risk Assessment Tool; OHT, Oral Health Therapist; PA, Public Act; PAR, Participatory Action Research; PHC, Primary Health Care; POHS, Preventive Oral Health Services; RACF, Residential Aged Care Facility; REDCap, Research Electronic Data Capture; RDHAP, Registered Dental Hygienist in Alternative Practice; SDF, Silver Diamine Fluoride; WIC, Special Supplemental Nutrition Program for Women, Infants, and Children.
